# Multigene phylogeny reveals a new Iranian earthworm genus (Lumbricidae: *Philomontanus*) with three new species

**DOI:** 10.1371/journal.pone.0208904

**Published:** 2019-01-30

**Authors:** Farnaz Bozorgi, Marjan Seiedy, Masoumeh Malek, Manuel Aira, Marcos Pérez-Losada, Jorge Domínguez

**Affiliations:** 1 School of Biology and Center of Excellence in Phylogeny of living organisms, College of Science, University of Tehran, Tehran, Iran; 2 Departamento de Ecoloxía e Bioloxía Animal, Universidade de Vigo, E-Vigo, Spain; 3 Computational Biology Institute, Milken Institute School of Public Health, George Washington University, Ashburn, VA, United Staes of America; 4 Department of Invertebrate Zoology, US National Museum of Natural History, Smithsonian Institution, Washington, DC, United States of America; 5 CIBIO-InBIO, Centro de Investigação em Biodiversidade e Recursos Genéticos, Vairão, Portugal; Nanjing Agricultural University, CHINA

## Abstract

Lumbricidae taxonomy is vastly restricted by the morphological simplicity of earthworms and their lack of complex appendices. This has led to confusing results in the Lumbricidae classifications, which in turn, has hindered our ability to identify and assign new and cryptic species to the family. Here we propose the addition of a new Lumbricidae genus from the Zagros and Elburz Mountains of Iran, i.e. *Philomontanus* gen. nov, including three new species. Our taxonomic inferences were based on the phylogenetic analysis of two nuclear gene regions (28S rDNA and 18S rDNA) and 11 mitochondrial gene regions (16S rDNA, 12S rDNA, NADH dehydrogenase I, cytochrome oxidase subunits I and II and tRNAs Asn, Asp, Val, Leu, Ala and Ser). *Philomontanus* gen. nov comprises the earthworm species *Philomontanus sarii* sp. nov., *Philomontanus mahmoudi* sp. nov. and *Philomontanus baloutchi* sp. nov. These three species are morphologically similar to each other with only a few characters separating them (e.g. size, pigmentation and position of clitellum). Our findings support the adoption of an integrative approach including molecular information (e.g., DNA sequences) to aid earthworm classification and develop a robust taxonomy.

## Introduction

Over the last years Iranian taxonomists have studied earthworms in different parts of Iran and published several studies [1, 2]. Those studies concluded that the Iranian earthworm fauna comprises at least 13 genera and 28 species, belonging to the families Lumbricidae, Megascolecidae and Acanthodrilidae [1, 2]. A recent survey by our group in two regions of Iran, the Zagros and Elburz Mountains, rendered 39 morphologically similar earthworm specimens that could be assigned to three morphotypes based on a few key characters (e.g., clitellum and tubercula pubertatis location and pigmentation). However, a comparative study of morphological diagnostic characters commonly used in earthworm taxonomy, was not sufficient to assign unequivocally these specimens to any known Lumbricidae genus. Delimitation of diagnostic morpho-anatomical features in earthworms has been hampered by high levels of homoplasy [3–6]. Recently, integrative approaches including molecular DNA sequences from multiple genes coupled with phylogenetic inference has been used to complement morphology-based taxonomic studies [4, 5, 7]. This approach has aided species delimitation, clarified taxonomic riddles and deciphered earthworm evolutionary relationships [4, 5, 7–16]. In the present study, we couple DNA sequences from multiple nuclear and mitochondrial gene regions and morphological evidence with phylogenetic analysis to determine the taxonomic position of three putative Lumbricidae earthworm species from two biologically diverse mountainous areas of Iran.

## Material and methods

### Taxon sampling

**Fig 1:**
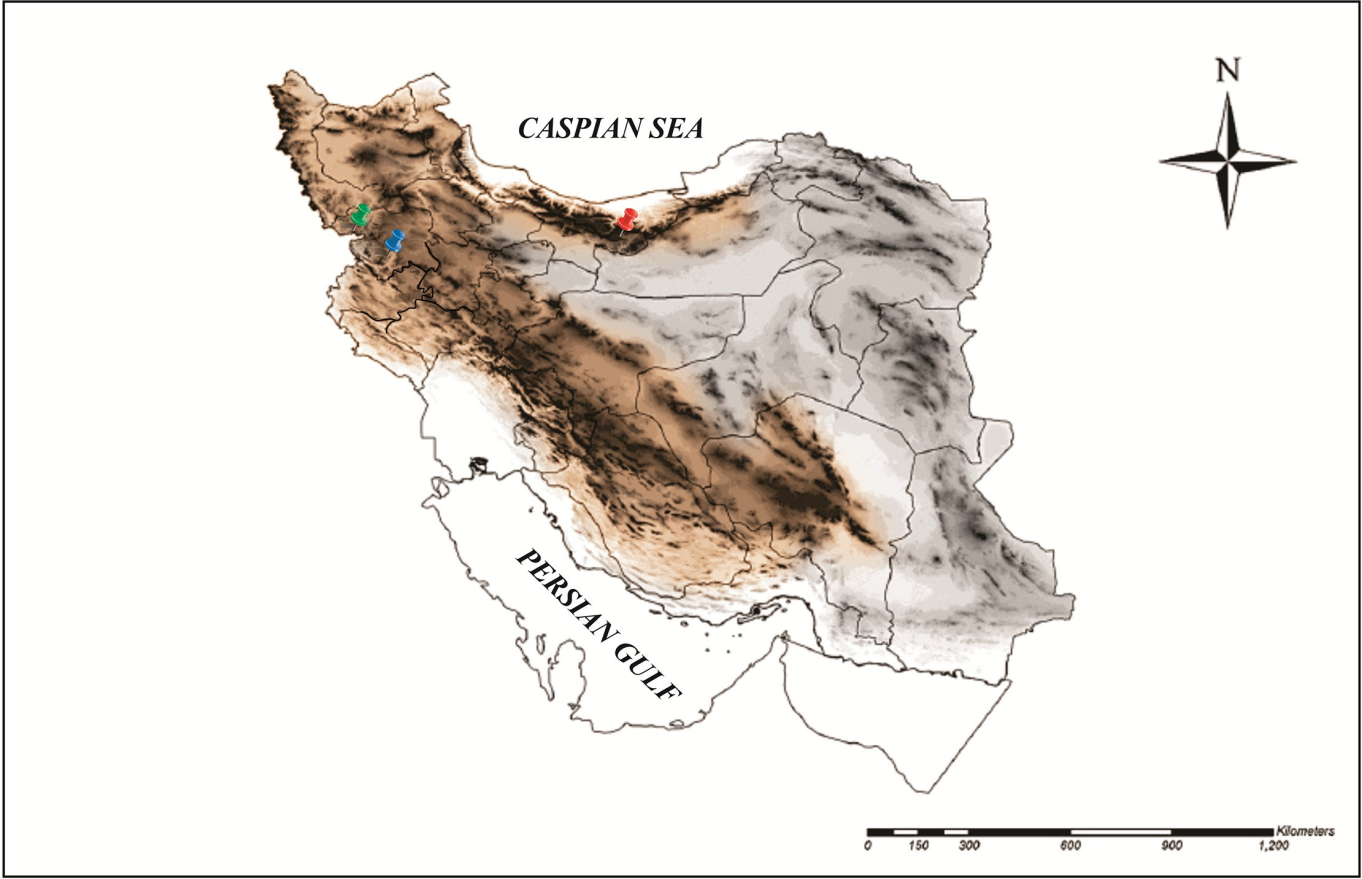
Study area in the Zagros and Elburz Mountains of Iran (This map is originally created by first author, using ArcGIS, Mapping & Analytical platform. Red, Type locality of *Philomontanus sarii* sp. nov, Elburz Mountains. Green, Type locality of *Philomontanus mahmoudi* sp. nov, Zagros Mountains. Blue, Type locality of *Philomontanus baloutchi* sp. nov, Zagros Mountains.

A total of 39 earthworm specimens were collected in June 2014 and July 2015 in two eco-regions of Iran, the Zagros and Elburz Mountains (Fig 1). The Elburz Mountains lie along the northern border of Iran at the southern shore of the Caspian Sea. Mountains dominate the landscape of this eco-region with mostly forest steppe [17]. We sampled the northern slopes, which have humid climate due to northerly airflow, enriched with moisture from the sea. This long mountain range forms a barrier between the Iran plateau (Northeast) and the Mesopotamian lowlands (Southwest), and also constitutes a corridor for the southward distribution of northern faunal elements [1]. Hence, the Elburz Mountains facilitate the distribution of species from northern to southern regions, and forms a barrier for species migration from eastern to western areas. The Zagros Mountains is part of the Alpine-Himalayan mountain range and begins in northwestern Iran and roughly spreads along the western border of the country [18]. The sampled Northern part of Zagros has sub-humid continental climate and winter precipitation.

We collected 23 earthworms in Mazandaran, Abbasabad, in the Elburz Mountains (36°36'29"N 51°07'38"E), and 10 and 6 specimens in Kurdistan, Baneh (36°02'15"N 45°56'50"E) and Kurdistan, Kamyaran (35°04'23"N 46°35'50"E), respectively, in the Zagros Mountains. Adult earthworms were collected by digging very small areas (<1 m^2^) and hand sorting, and then transferred to the laboratory. Earthworms were rinsed in water, anaesthetized in diluted ethanol, fixed in a mixture (1:1 v/v) of 90º ethanol and formaldehyde (4%) and placed in glass tubes containing 4% formaldehyde. Specimens and tissues collected for DNA extraction were preserved in absolute ethanol and stored at -20ºC. The species descriptions were made by external and internal visual examination using taxonomic identification keys [19–26]. Internal morphological examination was conducted by dorsal dissection under a stereomicroscope (Nikon SMZ1500). Body color was described in living specimens, whereas body dimensions were measured in fixed material. The following external and internal standard earthworm taxonomic characters were compared: size, number of body segments, body pigmentation, type of prostomium, arrangement of setae, presence and position of the first dorsal pore, nephridiopores, location and size of the male pores, type, start and end number of segments of clitellum, length and shape of tubercula pubertatis, segment and position of glandular tumescences, number and segment of testes, number of seminal vesicles, number of spermathecae, presence, location and extension of the gizzard and crop, segment and shape of typhlosole, segment of last pair of heart, extraoesophageals, excretory system, shape of nephridial bladder, shape of longitudinal muscle and presence of calciferous glands.

#### Ethics statement

None of the locations from which samples were collected required specific permissions. None of the earthworms collected in this study are listed as endangered or protected. All of the specimens included in this study are archived in the earthworm collection of the zoological museum at the University of Tehran with numbers 6572–6577 and are publicly accessible. The molecular data in this study are submitted into genbank with accession numbers LC427082-LC427098 and are publicly availbe.

### DNA extraction and phylogenetic analyses

Using the morphological characters above, we were able to split the collected specimens into three groups based on their geographic distribution and the following morphological differences: i) the Abbasabad group (23 specimens) from the Elburz Mountains: red violet, epilobic prostomium, size from 43–53 mm, 101–114 number of segments, clitellum in segments 25–30, tubercula pubertatis in segments 25–29; ii) the Baneh group (10 specimens) from the Zagros Mountains: totally pale, tanylobic prostomium, size from 25–31 mm, 51–70 number of segments, clitellum in segments 24–29, tubercula pubertatis in segments 25-28/29 and iii) the Kamyaran group (6 specimens) from the Zagros Mountains: red violet, epilobic prostomium, size from 57–68 mm, 123–140 number of segments, clitellum in segments 24–30, tubercula pubertatis in segments 26–29. We sequenced one specimen from each group.

**Fig 2:**
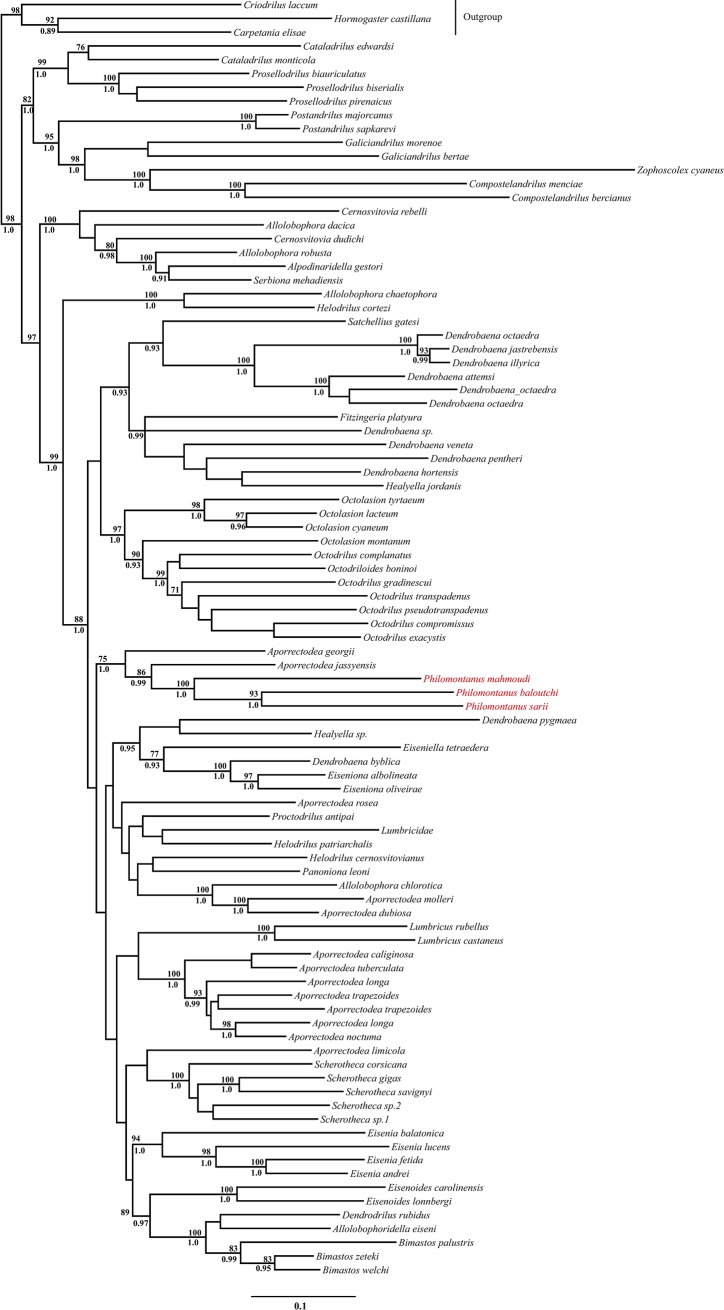
DNA maximum likelihood phylogenetic tree. Bootstrap proportions (if P>70%) and Bayesian posterior probabilities (if P>95%) are shown above and below the branches, respectively. The *Philomontanus* exemplars from each of the three morphological groups are shown in red.

One earthworm exemplar from each of the three morphological groups was used for the molecular and phylogenetic analyses. Total genomic DNA was extracted using the DNeasy Blood & Tissue Kit (Qiagen). Two nuclear gene regions (18S rDNA and 28S rDNA) and 11 mitochondrial gene regions (16S rRNA, 12S rRNA, NADH dehydrogenase (ND1), cytochrome oxidase subunit I and II (COI and COII) and tRNA Asn, Asp, Val, Leu, Ala and Ser) were amplified using the polymerase chain reaction (PCR), as previously described [4, 5, 7]. PCR products were purified using a MultiScreen PCRμ96 (Millipore) kit and sequenced bidirectionally using an Applied Biosystems (ABI) 377XL automated sequencer. The ABI Big-dye Ready-Reaction kit was used following the standard cycle sequencing protocol, but with a 16th of the suggested reaction size. DNA sequences were deposited in GenBank under Accession Numbers LC427082-LC427098. To determine the phylogenetic position of the Iranian taxa within the Lumbricidae, we performed a phylogenetic analysis of the new taxa and other available earthworm species in Domínguez et al. [7] and Pérez-Losada et al. [9]. This dataset included 89 ingroup species (Lumbricidae) and three outgroup (Hormogastridae and Criodrilidae) species (see Fig 2). Nucleotide sequences for each gene region (all tRNAs were combined into a single gene region) were aligned using MAFFT v6 [27, 28] under the global (G-INS-i) algorithm and default settings. Phylogenetic congruence among gene regions (COI: 658 bp, COII: 688 bp, 12S: 350 bp, 16S: 1408 bp, 18S: 781 bp, ND1: 929 bp, tRNAs: 708 bp, and 28S: 964 bp) was assessed using the protocol developed by Wiens [29]. No areas of strongly supported incongruence between gene trees were observed. Gene regions were analyzed in multiple concatenated partitions as in Domínguez et al. [7]. JModelTest v1.0.1 [30] was used to determine the best evolutionary model for each DNA partition under the Akaike Information Criterion (AIC) [31]. The general time reversible model [32], with invariable sites and gamma distribution, was selected for all partitions (GTR+G+I). Maximum likelihood analysis of the concatenated partitions was performed in RAxML v7.2.0 [33] with 1,000 replicates. Clade support was assessed using the non-parametric bootstrap procedure [34] with 5,000 bootstrap replicates. Bayesian analysis coupled with Markov chain Monte Carlo (BMCMC) inference was performed in Mr. Bayes v3.1.2 [35]. Four independent BMCMC analyses were run in the CIPRES Science Gateway portal [36], each consisting of four chains. Each Markov chain was started from a random tree and run for 2x10^7^ cycles, sampling every 1,000^th^ generation. Evolutionary model parameters were estimated independently for each data partition starting as unknown variables with uniform default priors. Convergence and mixing were monitored using Tracer v1.5 [37]. All sample points prior to reaching stationary levels were discarded as burn-in. The posterior probabilities for individual clades obtained from separate analyses were compared for congruence and then combined and summarized on a 50% majority-rule consensus tree.

#### Nomenclatural acts

The electronic edition of this article conforms to the requirements of the amended International Code of Zoological Nomenclature, and hence the new names contained herein are available under that Code from the electronic edition of this article. This published work and the nomenclatural acts it contains have been registered in ZooBank, the online registration system for the ICZN. The ZooBank LSIDs (Life Science Identifiers) can be resolved and the associated information viewed through any standard web browser by appending the LSID to the prefix "http://zoobank.org/". The LSID for this publication is: **urn:lsid:zoobank.org:pub:C9F380AF-B080-4CFE-A99A-F2539FC169B1.** The electronic edition of this work was published in a journal with an ISSN, and has been archived and is available from the following digital repositories: **PubMed Central, LOCKSS**.

## Results

### Phylogenetic inference

To determine the phylogenetic position of Iranian earthworms from Zagros and Elburz Mountains as well as their closest relatives we conducted a molecular phylogenetic analysis of a wide range of lumbricids and three non-Lumbricidae species (outgroup). Iranian earthworms fell in a well-supported clade within the Lumbricidae tree, depicted as (*Aporrectodea georgii*, *Aporrectodea jassyensis*, (*Philomontanus mahmoudi*, (*P*. *baloutchi*, (*P*. *sarii*)))) (Fig 2)–although formal descriptions are presented later in the paper, here we use the taxonomic names of the new taxa for clarity of interpretations. The Iranian clade was sister to a larger clade including the monophyletic lumbricid genera *Lumbricus*, *Scherotheca*, *Eiseniona*, *Eisenia* and *Eiseniodes* (Fig 2). The ML and Bayesian phylogenetic analyses showed strong support for the clade including the three Iranian species (bp = 100 and pP = 1.0), with the two species from Zagros Mountains forming a paraphyletic assemblage. The sister species to the Iranian clade, *Aporrectodea*. *jassyensis* and *A*. *georgii*, were collected in Turkey (Diyarbakir) and Serbia (Goc Mountains), respectively, suggesting a potential phylogeographic pattern of dispersion.

### Morphological differential diagnosis

The three new *Philomontanus* species are morphologically very similar with only some differences in size, pigmentation, type of prostomium, and length and position of clitellum and tubercula pubertatis (Table 1). Their main differences with their sister species, *A*. *georgii* and *A*. *jassyensis*, are: 1) the type of clitellum, which is saddle shaped for the two *Aporrectodea* species, 2) the setal arrangement, which is closely paired for two *Aporrectodea* species and 3) the shape of the nephridial bladders, which is J shape for two *Aporrectodea* species. Considering that the Iranian species formed a well-supported clade and were geographically separated and morphologically distinct from their closest relatives, i.e. *A*. *georgii* and *A*. *jassyensis*, we have adopted an inclusive taxonomic approach to revise the current Lumbricidae classification. Hence, we propose that the three Iranian earthworm species should be placed in a new genus *Philomontanus* (including *P*. *sarii*, *P*. *mahmoudi* and *P*. *baloutchi*). Further sampling and phylogenetic analyses of earthworms from these contiguous geographical areas may suggest new splits, but at this point, we opt for a more conservative approach.

**Table 1:** Morphological characters compared between the three *Philomontanus* earthworm species under study. * = Morphological differences.

	Species		
Morphological characters	*Philomontanus sarii* sp. nov.	*Philomontanus mahmoudi* sp. nov.	*Philomontanus baloutchi* sp. nov.
*Size	43–53 mm	25–31 mm	57–68 mm
*Number of segments	101–114	51–70	123–140
*Pigmentation	Red violet	Totally pale	Red violet
Setae arrangement	Widely distant	Widely distant	Widely distant
Clitellum	Annular	Annular	Annular
*Segment of clitellum	25–30	24–29	24–30
Tubercula pubertatis	Ribbon shape	Ribbon shape	Ribbon shape
*Segment of tubercula pubertatis	25–29	25-28/29	26–29
*Prostomium	Epilobic	Tanylobic	Epilobic
First Dorsal pore	visible on 7/8	visible on 7/8	visible on 7/8
Male pore	Hardly seen on segment 15	Hardly seen on segment 15	Hardly seen on segment 15
Nephridiopores	Hardly seen, *b-c*	Visible, *b-c*	Visible, *b-c*
Septa	Visible, strengthened in 6/7, 8/9 and 11/12	Visible, no recognizable strengthened	Visible, strengthened in 7/8, 9/10 and 14/15
		Species	
Morphological characters	*Philomontanus sarii* sp.nov.	*Philomontanus mahmoudi* sp.nov.	*Philomontanus baloutchi* sp.nov.
Testes	Two pairs, segments 10–11	Two pairs, segments 10–11	Two pairs, segments 10–11
Seminal vesicles	Four pairs, segments 9–12	Four pairs, segments 9–12	Four pairs, segments 9–12
Spermathecae	Two pairs, segments 9/10-10/11, *c-d*	Two pairs, segments 9/10-10/11, c-d	Two pairs, segments 9/10-10/11, c-d
Calciferous glands	Visible, segment 11	Visible, segment 11	Visible, segment 11
Crop location	15–16	15–16	15–16
Gizzard location	17–18	16–17	17–18
Typhlosole	Simple lamelliform, quite large	Simple lamelliform, quite large	Simple lamelliform, large
Last pair of heart	11	11	11
Extraoesophageals	12	12	12
Excretory system	Holonephridial	Holonephridial	Holonephridial
Nephridial bladder	Ocarina shaped	Ocarina shaped	Ocarina shaped
Longitudinal muscle	Pinnate type	Pinnate type	Pinnate type
Glandular tumescences	9–11, *a-b*	9–11, a-b	9–11, a-b

### Taxonomy

Phylum Annelida Lamarck, [38]

Class Oligochaeta Grube, [39]/ Clitellata Michaelsen, [40]

Order Megadrilli Benham, [41] /Haplotaxida Michaelsen, [42]

Family Lumbricidae Rafinesque-Schmaltz, 1815[43]

### Genus *Philomontanus* gen.nov.urn:lsid:zoobank.org:act: 95F2ACE7-5D0E-4691-BFD5-A455D85214BB

Diagnosis: Setae widely distant. Pigmentation variable, red-violet or totally pale. Prostomium epi- or tanylobous. First dorsal pore around 7/8. Male pore on 15 very small, on setal line *bc*. Clitellum annular. Spermathecae, which are small and round shaped present, frequently empty in 9/10–10/11 and open in setal line *cd*. Tubercula pubertatis ribbon shape. Nephridiopores aligned in setal *bc*. Two pairs of testes and funnels in segments 10–11, not iridescent. Four pairs of seminal vesicles in 9–12, those in 11 and 12 are slightly smaller than others. Two pairs of small, roundly shaped and almost empty spermathecae in 9/10 and 10/11 and open in setae line *cd*. Calciferous glands in segments 11 with hardly recognizable diverticula. Excretory system holoic, nephridial bladders ocarina shape. Typhlosole simple lamelliform, the cross-section of longitudinal muscle layer is of pinnate type. Since the morphological characters above are not sufficient to pinpoint the taxonomic position of *Philomontanus* in the Lumbricidae, we have used phylogenetic analysis of DNA sequences to determine its taxonomic position and closest relatives.

Type species: *Philomontanus sarii* sp. nov. by original designation herein.

Included species: *Philomontanus sarii* sp. nov., *P*. *mahmoudi* sp. nov. and *P*. *baloutchi* sp. nov.

Etymology. *Philomontanus* is composed of *Philo* (Greek prefix means liking for specified thing) and *montanus* (Latin noun means mountains). This name has been chosen as new genus and its three species have been found in mountains.

Remarks. The closest genus based on morphological evidence is *Dendrobaena* [44], but with distinguishable differences in pigmentation (reddish vs. both reddish and pale), size and number of body segments (small to medium sized species vs. larger range of size), shape of clitellum (saddle shape vs. annular shape) and shape of tubercles (different shapes observed; nob shape, ribbon shape and etc. vs. ribbon shape). However, according to morphological descriptions in several references, it is obvious that *Dendrobaena* is very heterogeneous [45–47], not monophyletic and needs taxonomic revision [7, 16]. The annular clitellum could relate *Philomontanus* to *Eiseniona albolineata* or to *Bimastos*, although our phylogenetic analyses showed otherwise. According to their morphological features and characteristics of the habitat where they were sampled, the *Phylomontanus* species could be considered epigeic earthworms.

**Fig 3:**
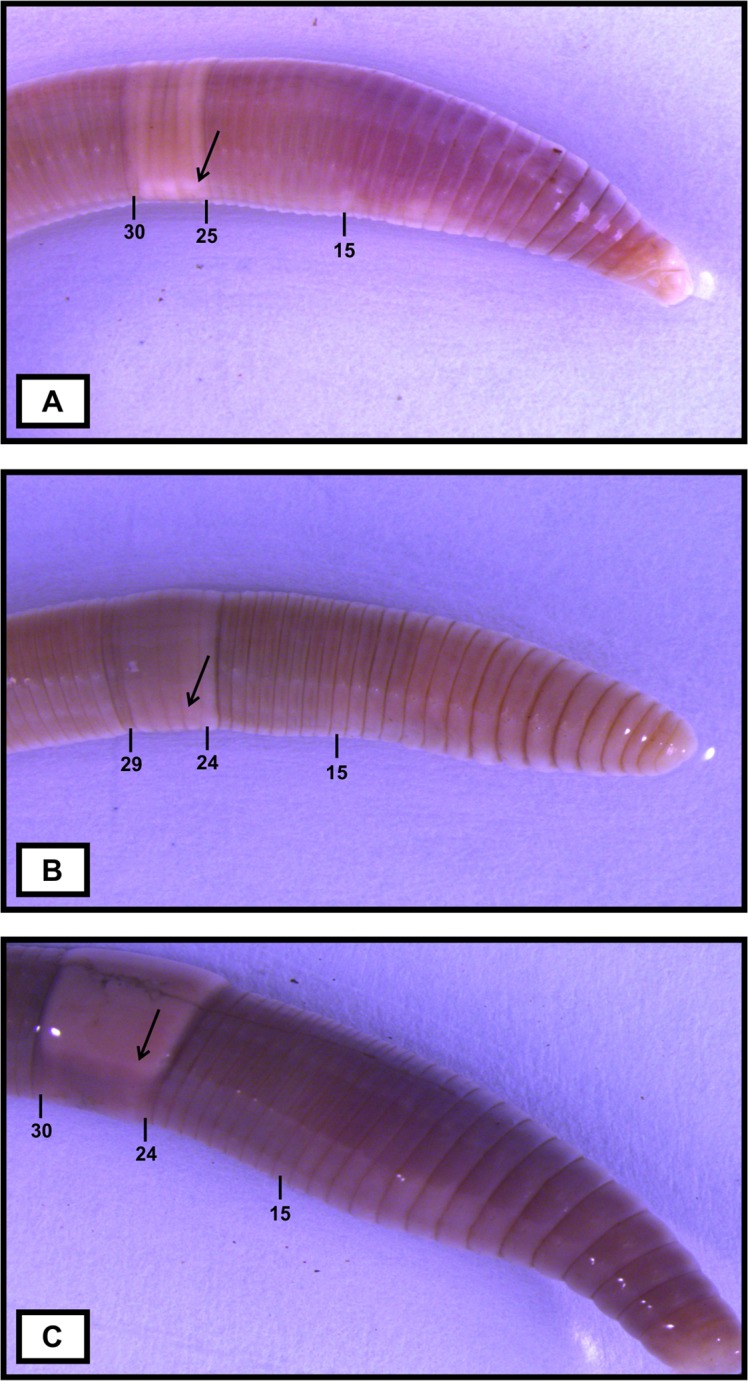
Ventrolateral view of the *Philomontanus* species. Arrows point to tubercula pubertatis A. *Philomontanus sarii* sp. nov. B. *Philomontanus mahmoudi* sp. nov. C. *Philomontanus baloutchi* sp. nov..

**Fig 4:**
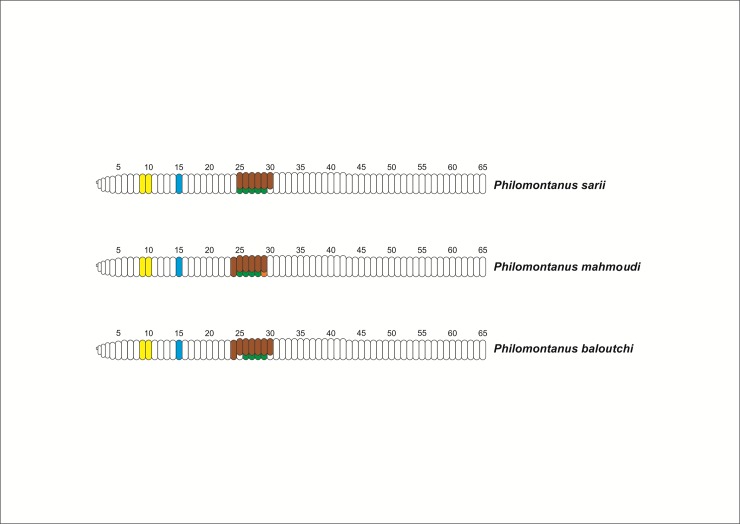
Diagram of external morphology of the *Philomontanus* species. Yellow, Spermathecae Blue, Male pore Brown, Clitellum Green, Tubercula pubertatis Orange, Variation of Tubercula pubertatis.

***Philomontanus sarii* Bozorgi & Malek sp. nov.** urn:lsid:zoobank.org:act:2F35B21F-9764-4AFA-8EBB-75DDD54147FD. Figs 3 and 4

Etymology. The new species name was chosen to honor Dr. Alireza Sari, who has done extensive research on various aspects of Iranian fauna.

**Fig 5:**
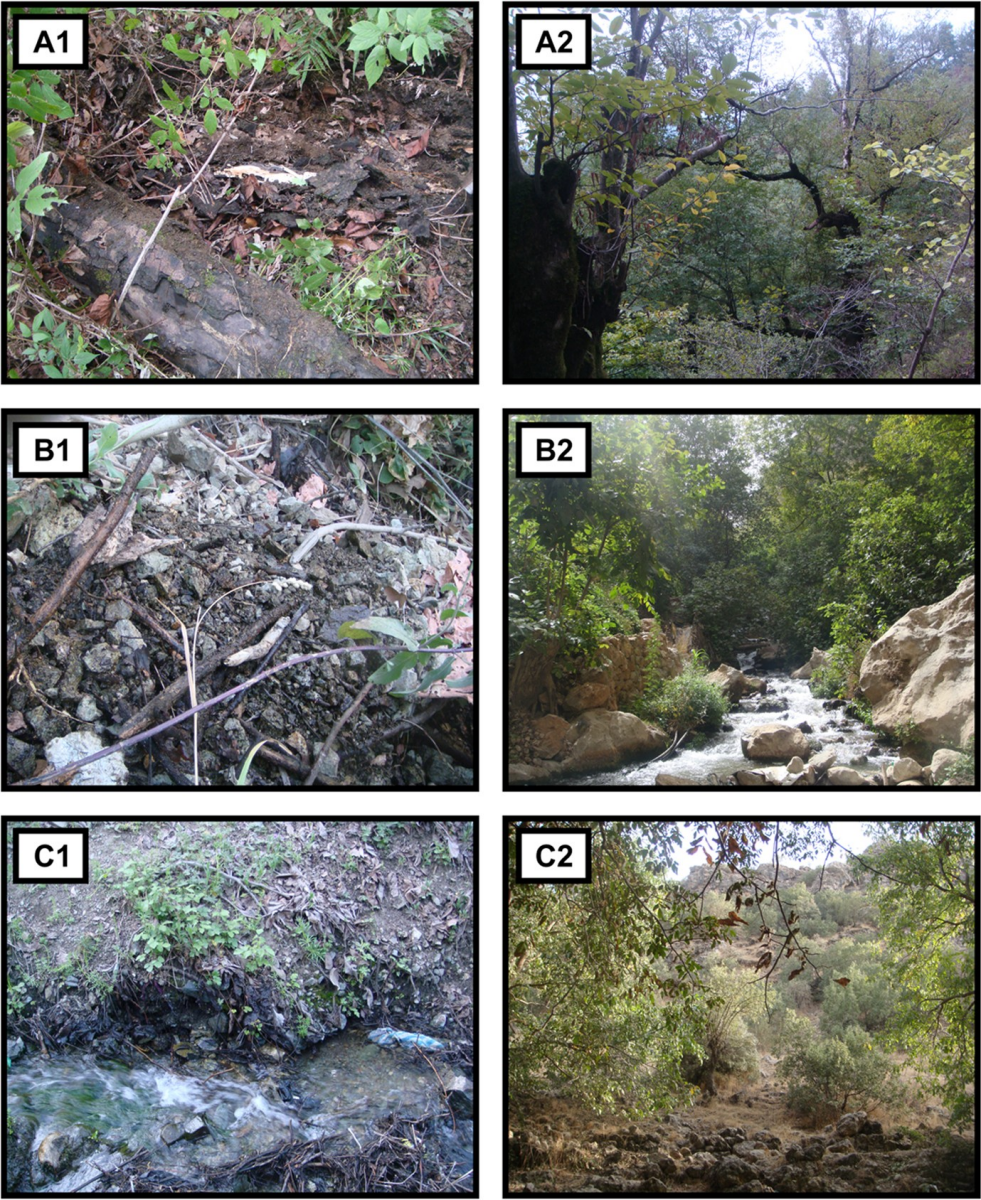
Sampling sites of new species. (A1, A2) Mazandaran, Abbasabad (36.72 ^o^ N; 51.11^o^ E), Elburz Mountains, sampling site of *Philomontanus sarii* sp. nov. (B1, B2) Kurdistan, Kamyaran (35.49^o^ N; 46.35^o^ E), Zagros Mountains, sampling site of *Philomontanus baloutchi* sp. nov. (C1, C2) Kurdistan, Baneh (35.99^o^ N; 45.88^o^ E), Zagros Mountains, sampling site of *Philomontanus mahmoudi* sp.nov.

Sampling site. Oak (*Quercus* sp.) forest near mountain hillside, semi humid brown soil, covering in decaying plants and rotting logs on the forest floor. Plants present: *Juniperus* sp., *Pistacia* sp. and *Acer* sp. (Fig 5).

Type material. **Holotype,** ZUTC/ 6572, Abbasabad, Mazandaran, Elburz mountains, Iran (36°36'29"N 51°07'38"E). Leg. F. Bozorgi and A. Kazemi, 02.06.2014. Deposited in the earthworm collection of the Zoological Museum, University of Tehran. **Paratypes**, ZUTC/ 6573, 22 ex., Same locality and collection date as the Holotype. Leg. F. Bozorgi and A. Kazemi. Deposited in earthworm collection of Zoological Museum, University of Tehran.

Diagnosis. Body length 45–53 mm, diameter 2.8–3.1 mm. Color red-violet (Fig 3). Prostomium epilobous, first dorsal pore in 7/8. Setae widely distant, glandular tumescences visible in 9–11,ab. Clitellum annular, on segments 25–30. Male pores hardly seen on 15. Nephridiopores hardly seen between *b* and c. Septa visible and strengthened in 6/7,8/9 and 11/12. Calciferous glands in segment 11 with no diverticula. Excretory system holoic. Nephridial bladders ocarina shape. Typhlosole simple lamelliform, the cross-section of longitudinal muscle layer is of pinnate type.

Description. Length of the holotype 53 mm, diameter just after the clitellum 3.1 mm. Number of segments 114. Paratypes 43–53 mm long and 2.8–3.1 mm wide. Number of segments 101–114. Color preserved dorsal red violet pigmented, less dense of pigmentation ventrally (Fig 3). Prostomium epilobous. First dorsal pore in the intersegmental furrow 7/8. Setae widely distant. Setal formula at segment 20; aa:ab:bc:cd:dd = 1.50:1.25:1.11:1:2.3. Male pores hardly seen, in very small size on the segment 15 between setae *bc*. Nephridiopores hardly seen aligned in setal line *bc*. Clitellum on 25–30 annular. Tubercula pubertatis ribbon shape on 25–29. Glandular tumescences on segments 9–11*ab*. Septa thickened in 6/7, 8/9 and 11/12. Two pairs of testes and funnels in segments 10–11, not iridescent. Four pairs of seminal vesicles in 9–12, those in 11 and 12 are slightly smaller than others. Two pairs of small, roundly shaped and almost empty spermathecae in 9/10 and 10/11 and open in setae line *cd*. Calciferous glands in11. Last pair of hearts in 11. Excretory system holonephridial. Nephridial bladders ocarina- shaped with an intangible bump at the end. Crop in 15–16, gizzard in 17–18. Typhlosole simple lamelliform, quiet large. Longitudinal muscle layer of pinnate type.

Remarks. This new species is morphological similar to *Dendrobaena byblica* (Rosa, 1893), but with slight differences in size (22-42mm vs. 43-53mm), segment of clitellum (23/24-29 vs. 25–30), shape of tubercula pubertatis (verity shapes vs. ribbon shape) and position of tubercula pubertatis (25–27 vs. 25–29).

***Philomontanus mahmoudi* Bozorgi & Malek sp. nov.** urn:lsid:zoobank.org:act:B3BF1AEB-D20C-43B5-84AB-E42C4C83125F. Figs 3 and 4

Etymology. The new species is named in memory of coauthor Masoumeh Malek’s father.

Sampling site: Walnut (*Juglans regia*) forest near mountain slope, semi humid soil with carious plants and gravel developed over slate. Plants present: *Crataegus* sp., *Pistacia* sp. and *Quercus* sp. (Fig 5).

Type material. **Holotype,** ZUTC/ 6574, Baneh, Kurdistan, Zagros Mountains, Iran (36°02'15"N 45°56'50"E). Leg. F. Bozorgi, 13.07.2015. Deposited in the earthworm collection of the Zoological Museum, University of Tehran. **Paratypes**, ZUTC/ 6575, 9 ex., Same locality and collection date as the Holotype. Leg. F. Bozorgi. Deposited in earthworm collection of Zoological Museum, University of Tehran.

Diagnosis. Body length 25–31 mm, diameter 2.1–2.5 mm. Color totally pale (Fig 3). Prostomium tanylobous, first dorsal pore in 7/8. Setae widely distant, glandular tumescences visible in 9–11,ab. Clitellum annular, on segments 24–29. Male pores hardly seen on 15, nephridiopores visible between *b* and c. Septa visible with no recognizable strengthened. Calciferous glands in segment 11 with slight diverticula. Excretory system holoic. Nephridial bladders ocarina shape. Typhlosole simple lamelliform, quiet large, the cross-section of longitudinal muscle layer is of pinnate type.

Description. Length of the holotype 27 mm, diameter just after the clitellum 2.4 mm. Number of segments 67. Paratypes 25–31 mm long and 2.1–2.5 mm wide. Number of segments 51–70. Color totally pale without any pigmented parts all over the body (Fig 3). Prostomium tanylobous. First dorsal pore in the intersegmental furrow 7/8. Setae widely distant. Setal formula at segment 20; aa:ab:bc:cd:dd = 1.20:1.12:1:1.05:1.5. Male pores hardly seen, in very small size facing ventral on the segment 15 between setae *bc*. Nephridiopores aligned in setal line *bc*. Clitellum on 24–29 annular. Tubercula pubertatis ribbon shape with more swelling in some individual on 25–28/29. Glandular tumescences on segments 9–11*ab*. Septa seen but there were no recognizable difference for thickness between segments. Two pairs of testes and funnels in segments 10–11, not iridescent. Four pairs of seminal vesicles in 9–12, those in 11 and 12 are slightly smaller than others. Two pairs of small, roundly shaped and almost empty spermathecae in 9/10 and 10/11 and open in setae line *cd*. Calciferous glands in 11. Last pair of hearts in 11. Excretory system holonephridial. Nephridial bladders ocarina- shaped with little more elongation. Crop in 15–16 and gizzard in 17–18. Typhlosole simple lamelliform, quiet large. Longitudinal muscle layer of pinnate type.

Remarks. This new species has also morphological resemblance with *P*. *sarii* but differences in size (43–53mm vs. 25–31 mm), pigmentation (red violet vs. pale), position of clitellum (25–30 vs. 24–29) and position of gizzard (17–18 vs. 16–17).

***Philomontanus baloutchi* Bozorgi & Malek sp. nov.** urn:lsid:zoobank.org:act:473B8329-9E95-44CA-8A22-4CA20F4E2F2B. Figs 3 and 4

Etymology. The new species is named in memory of Dr. Mohammad Baloutch, who was a prominent zoology professor in Iran.

Sampling site: Walnut (*Juglans regia*) forest near mountain springs with cool slow flowing water, in the steep slope of valleys with permanent slow water, humid loam soil covered with carious plants. Plants present: *Crataegus* sp., *Pistacia* sp. and *Quercus* sp. (Fig5).

Type material. **Holotype,** ZUTC/ 6576, Kamyaran, Kurdistan, Zagros Mountains, Iran (35°04'23"N 46°35'50"E). Leg. F. Bozorgi, 23.07.2015. Deposited in the earthworm collection of the Zoological Museum, University of Tehran. **Paratypes**, ZUTC/ 6577, 5 ex., Same locality and collection date as the Holotype. Leg. F. Bozorgi. Deposited in earthworm collection of Zoological Museum, University of Tehran.

Diagnosis. Body length 57–68 mm, diameter 3.8–4.7 mm. Color red-violet (Fig 3). Prostomium epilobous, first dorsal pore in 7/8. Setae widely distant, glandular tumescences visible in 9–11, ab. Clitellum annular, on segments 24–30. Male pores hardly seen on segment 15, nephridiopores visible between *b* and c. Septa visible, strengthened in 7/8, 9/10 and 14/15. Calciferous glands in segment 11. Excretory system holoic. Nephridial bladders ocarina shape. Typhlosole simple lamelliform, large, the cross-section of longitudinal muscle layer is of pinnate type.

Description. Length of the holotype 67 mm, diameter just after the clitellum 4.2 mm. Number of segments 136. Paratypes 57–68 mm long and 3.8–4.7 mm wide. Number of segments 123–140. Red violet pigmented, less dense pigmentation ventrally especially on primary segments. (Fig 3). Prostomium epilobous. First dorsal pore in the intersegmental furrow 7/8. Setae widely distant. Setal formula at segment 20; aa:ab:bc:cd:dd = 1.21:1.14:1:1.03:1.5. Male pores hardly seen, in very small size on the segment 15 between setae *bc*. Nephridiopores aligned in setal line *bc*. Clitellum on 24–30 annular. Tubercula pubertatis ribbon shape on 26–29. Glandular tumescences on segments 9–11*ab*. Septa visible, strengthened in 7/8, 9/10 and 14/15. Two pairs of testes and funnels in segments 10–11, not iridescent. Four pairs of seminal vesicles in 9–12, those in 11 and 12 are slightly smaller than others. Two pairs of small, roundly shaped and almost empty spermathecae in 9/10 and 10/11 and open in setae line *cd*. Calciferous glands in 11. Last pair of hearts in 11. Excretory system holonephridial. Nephridial bladders ocarina- shaped. Crop in 15–16. gizzard in 17–18. Typhlosole simple lamelliform, large. Longitudinal muscle layer of pinnate type.

Remarks. This new species also has some morphological similarity to *D*. *byblica* (Rosa, 1893), but with differences in size (22–42 mm vs. 57–68mm), position of clitellum (23/24-29 vs. 24–30), shape of tubercles (verity shapes vs. just ribbon shape) and position of tubercles (25–27 vs. 26–29). This new species differs from *P*. *sarii* in size (43-53mm vs. 57-68mm), position of clitellum (25–30 vs. 24–30) and position of tubercles (25–29 vs. 26–29). It also differs from *P*. *mahmoudi* in size (25–31 mm vs. 57–68mm), pigmentation (pale vs. red violet), position of clitellum (24–29 vs. 24–30), position of tubercles (25–28/29 vs. 26–29) and position of gizzard (16–17 vs. 17–18).

## Discussion

The accurate delimitation of species boundaries is essential to numerous areas of biological research, especially for the belowground fauna, a major component of global biodiversity [47]. The classification of earthworms at the genus level in the family Lumbricidae has always been puzzling and varies according to different authors, with proposals ranging from 6 to 45 genera [7]. Moreover, those classifications are based on lists of diagnostic morphological characters that are mostly simple structures and can vary and overlap among taxa [4]. Phylogenetic analysis of DNA sequences is a powerful systematic tool to assess biodiversity, identify new species and untangle cryptic diversity [48]. Hence, using molecular-based phylogenies to investigate earthworm systematics has become common practice for researches nowadays [49]. In this study, multilocus phylogenetic inference and morphological evidence were used to identify and describe three new earthworm species from the Elburz and Zagros Mountains in Iran. The 39 collected specimens were morphologically similar but could be confidently assigned to three different morphotypes based on differences in a few characters (like clitellum and tubercula pubertatis position and pigmentation). The standard diagnostic characters used in earthworm taxonomy were similar to those seen in several Lumbricidae genera, hence it was impossible to unequivocally assign them to any known Lumbricidae genus. Our phylogenetic analyses of three Iranian exemplars showed, however, three divergent lineages in a clade clearly separated from the other Lumbricidae genera and well-supported by our bootstrap (BP> 70%) and Bayesian (PP> 0.95) analyses. This, hence, suggests that the Iranian specimens correspond to three new congeneric species and should be included in a new Lumbricidae genus, *Philomontanus* gen. nov. Our phylogenetic analysis also indicates that the sister taxa to *Philomontanus* spp. are *A*. *jassyensis and A*.*georgii*. According to Domínguez et al. [7], *A*. *jassyensis and A*.*georgii* are not valid species within the *Aporrectodea* genus and need to be re-classified within the Lumbricidae. Qiu and Bouché [19] created the genus *Koinodrilus* for *K*. *georgi*, K. *jasseyensis* as well as other earthworm species. However, Czusdi and Zicsi [25] and Blakemore [26] clearly indicated that *Koinodrilus* is a synonymous of *Aporrectodea*. Therefore, the external diagnosis of the genus *Aporrectodea* is setae closely paired, pigmentation lacking and prostomium epilobic. These morphological features are not seen in *Philomontanus*, which includes small and medium-sized reddish or pale lumbricids, with prostomium type epilobic or tanylobic and widely distant setae. Hence, given all this evidence, we consider that *Phylomontanus* is a valid genus morphologically distinct from *Aporrectodea* or the obsolete *Koinodrilus* genus.

Previous earthworm species collected in Iran were not preserved adequately to carry out molecular systematics comparisons, hence their evolutionary relatedness to the new genus described here could not be assessed. Additionally, no previous studies exist describing the phylogenetic radiation of Iranian lumbricids.

Earthworm systematics and taxonomy can benefit greatly from the integration of morphological and molecular lines of evidence. Preliminary morphology-based taxon identifications in the field or laboratory can be then re-evaluated using DNA barcoding genes (COI) or multigene phylogenies. In many earthworm groups, species boundaries are blurred, which makes global estimates of diversity difficult and sometimes unrealistic [50, 51]. Additionally, conservation efforts aiming at preserving the evolutionary potential of a species, can greatly benefit from this integrative approach by assessing the phylogenetic diversity of the species under consideration [52]. We encourage earthworm taxonomists to combine morphological and molecular evidence with available ecological and geographical information to investigate Lumbricidae systematics. Such an integrative approach will most certainly provide a powerful tool for identifying new and cryptic earthworm species and will lead to a better understanding of their diversity and the processes maintaining it.
